# Special Issue: Characterization of Innovative Asphalt Materials for Use in Pavement Design and Analysis

**DOI:** 10.3390/ma15051883

**Published:** 2022-03-03

**Authors:** Gilda Ferrotti, Andrea Graziani

**Affiliations:** Department of Civil and Building Engineering and Architecture, Università Politecnica delle Marche, 60131 Ancona, Italy; a.graziani@staff.univpm.it

## 1. Introduction

The development of innovative and sustainable materials for use in asphalt pavement applications has received increasing attention over the past 20 years, also thanks to the growing interest in the circular economy approach, which is replacing the linear one [1,2,3,4,5,6]. High-performance [7,8,9] and/or innovative materials such as nanomaterials for asphalt modification [9,10,11] can be employed for investigating the possibility of achieving a longer service life of pavement structures, maximizing the material productivity. At the same time, the use of renewable materials in partial replacement of asphalt, such as bio-binders [12,13,14,15], and the recycling of end-of-life pavement materials with hot [16,17], warm [18,19] and cold [20,21,22] technologies is becoming more and more popular nowadays. However, these innovative/high-performance materials can be used for paving mixtures only after checking their ability to resist both traffic and environmental actions. Moreover, bringing these innovations from laboratory-scale studies to industrial or pavement-scale applications is now an open challenge that can only be addressed through a rigorous performance- and design-related characterization.

Accordingly, this Special Issue of *Materials* aimed at collecting research focused on the development of innovative and sustainable asphalt materials, as well as on mixture and structural design. With the aim of seeking the right material to solve market-driven challenges, the following aspects were investigated:

- Performance evaluation of innovative materials, such as renewable materials and graphite nanoplatelets;
- Investigation of the possible employment of wastes (sanitary ceramics, crumb rubber, reclaimed asphalt) in asphalt pavement applications.

The research performed by numerous universities and scientific centres from Europe, Asia and North America demonstrated that innovative/high-performance materials can be employed in asphalt pavement applications only if their behaviour is comparable to that of the conventional asphalt materials, for which the design approaches were originally conceived and validated.

## 2. Short Description of the Articles Published in This Special Issue

Twenty universities and scientific centres contributed with their original research papers to this Special Issue of *Materials*. The issues covered can be divided into five main topics:

- Characterization of recycled materials in asphalt concrete mixtures, such as waste of sanitary ceramics [23], crumb rubber (CR) from waste tires [24,25,26] and reclaimed asphalt (RA) [27,28];
- Investigation of natural [28] or bio-based [29] materials used as rejuvenators in recycled asphalt applications;
- Study of rheological and fatigue behaviour of bituminous materials with [30] and without [31] the use of renewable bio-materials;
- Evaluation of lubricant characteristics of graphite nanoplatelets [32];
- Fatigue life prediction model for materials employed on long-span steel bridge decks [33].

Andrzejuk et al. [23] evaluated the possibility of recycling aggregates from waste sanitary ceramics in asphalt concrete mixtures. The mineral composition, the physical and mechanical properties, the morphology and the texture of the aggregates, as well as the affinity between aggregate and bitumen were investigated. They observed that the recycled aggregates provided sufficient properties regarding grain shape, freezing and thawing resistance and resistance to fragmentation. They also noted insufficient affinity between bitumen and aggregates by suggesting, in future works, the use of selected adhesion agents for improving this property.

Zhang et al. [24] investigated the effectiveness of different methods for modifying a base bitumen with CR: pre-treatment of the CR particles by microwave activation, use of the warm mix additive Sasobit and trans-polyoctenamer (TOR) procedure. Physical properties (softening point, elastic recovery, rotational viscosity) and rheological tests (frequency sweep, multiple stress creep and recovery) were performed. The trans-polyoctenamer (TOR) procedure provided higher temperature stability, elastic recovery and viscosity with respect to the other two methods. On the contrary, the microwave-activated rubberized bitumen had the lowest viscosity, but also the worst performance at high temperature and in terms of elastic recover. The addition of Sasobit to the rubberized bitumen provided intermediate performance with respect to the other two methods.

The study presented by Gawdzik et al. [25] was aimed at investigating the process of modification of bitumen with crumb rubber, paying particular attention to the rubber addition percentages (10 and 15%), mixing times (2, 4 and 8 h) and mixing temperatures (190 and 220 °C). The results showed that both temperatures improved the properties of the base bitumen, although the bitumen modified at 190 °C contained more non-degraded rubber. The increase of the mixing time led to the dissolution of the rubber crumbs and to its de-vulcanization. Moreover, it has been shown that bitumens modified in this way were characterized by high storage stabilities.

The aim of the research presented by Li et al. [26] concerned the possibility of preparing disintegrated high-volume crumb rubber asphalt (DHVRA) by adding a disintegrating agent in the asphalt rubber preparation process. This new procedure tried to solve the problems related to the traditional production method of the asphalt rubber (AR) by maximizing the recycling of waste CR and obtaining a homogeneous AR with low viscosity and good storage stability and improving its poor low-temperature performance. To this aim, they analysed the micro-disintegrating mechanism and evaluated high-temperature and low-temperature rheological properties of DHVRA. Results indicated that with an optimum dosage of disintegrating agent it is possible to promote the melt decomposition of CR, to reduce the viscosity and to improve the storage stability of DHVRA.

In the paper presented by Ferrotti et al. [27] a trial pavement section, prepared with a cold recycled asphalt mixture (with 88% RA) laid as binder course, was monitored for more than two years. The comparison between field curing and oven-curing in laboratory at 40 °C was performed in terms of indirect tensile strength, complex modulus, air voids content, as well as evolution of indirect tensile stiffness modulus as a function of curing time. Results showed that the development of material stiffness can be accelerated with a small effect on its long-term value if oven-curing is applied a few days/weeks after compaction. Moreover, the complex modulus values confirmed that cold recycled asphalt mixtures provide an intermediate behaviour between asphalt concrete mixtures and cement-treated mixtures.

The research performed by Slabonski et al. [28] concerned the investigation of the properties of a rejuvenator (main active components of natural origin) used in asphalt concrete mixtures with low (20%), medium (40%) and high (100%) percentages of RA. Both laboratory and field trial specimens were tested, as well as the warm mix asphalt (WMA) technology. The achieved results showed that the rejuvenator improved the homogenization of RA with the asphalt binder and aggregate in each mixture type. Suitable compaction characteristics were obtained for WMA mixtures with an increased content of RA.

Nciri [29] et al. studied the performance of a bio-based rejuvenator derived from waste pig fat (WPF) used in recycled asphalt applications. Different dosages of WPF (0, 3, 6, and 9% by binder weight) were added to a binder recovered from a RA for producing bitumen blends that were investigated in terms of chemical and rheological properties. Results showed that this rejuvenator can rehabilitate the RA binder that was compromised during the aging process, reduce the binder mixing and compaction temperatures, assist in adjusting the properties of the recovered binder by reducing viscosity and softening point values and increasing penetration values. Moreover, it can improve low-temperature performance and resistance to fatigue cracking, even if it can reduce the rutting resistance and the high-temperature performance.

The research presented by Gaudenzi et al. [30] involved rheological measurements aimed at evaluating the fatigue behaviour and comparing the self-healing capability of two plain bitumen and a bio-binder obtained by partially replacing one of the plain bitumen with a renewable bio-oil. They investigated the effects of bitumen type, amount of bio-oil addition, and aging on the healing potential of binders, evaluated by means of a recently proposed approach. Results showed that the method for healing analysis is suitable for both conventional and bio-add binders and that the fatigue and self-healing characteristics are mainly dependent on binder consistency but are also affected by aging. Moreover, the addition of bio-oil may increase the healing potential with respect to conventional bitumen, especially in aged condition. These results encourage the use of such bio-binders in road applications to obtain significant benefits in terms of performance and durability.

The aim of the research presented by Al-Mohammedawi et al. [31] concerned the influence of active fillers (i.e., cement) on rheological properties and fatigue behaviour of cold bitumen emulsion (CBE) mastic. Both chemical and rheological analysis were performed on mastics prepared with a bituminous emulsion and seven different fillers. Results showed that the rheological performance and the fatigue damage resistance depend not only on the filler inclusions, but also on filler type and chemistry. For specific fillers, the rise in the norm of the complex shear modulus and the decrease in the viscous component is associated with a significant enhancement in fatigue performance.

The objective of the study performed by Yan et al. [32] was to examine the effect of graphite nanoplatelets (GNP) on the lubricating behaviour of asphalt binders, trying to correlate the improved compactability observed for asphalt mixtures modified with GNP with the lubrication properties of the GNPs modified binder. Three binders with different percentages of GNP (0, 3 and 6% by binder weight) and different substrates (smooth and rough to simulate the mineral aggregate surface micro-texture) were investigated using viscosity and tribological tests. Results showed that, in the case of smooth substrates, GNP do not improve the lubricating behaviour of the binder. On the contrary, when rough substrates (which better represent the aggregate surface) are investigated, the lubrication properties of the binder are progressively improved as the GNP amount increases. Thus, the addition of GNP can enhance the lubrication properties of the binder when mixed with mineral aggregates confirming that the viscosity is not the only parameter involved in the compaction of asphalt mixtures, as the interaction between the aggregates plays a crucial role.

In the paper presented by Xu et al. [33], a fatigue life prediction model for asphalt concretes laid on long-span steel bridge decks was established based on the mechanism of fatigue damage evolution of materials from the microscopic perspective. Moreover, the reliability of the model was verified by fatigue tests performed on epoxy asphalt (EA), stone matrix asphalt (SMA) and gussasphalt (GA). The proposed model can provide a reference to predict the fatigue life of steel–asphalt concrete composite decks with cracked or non-cracked asphalt concrete layers. Epoxy asphalt showed higher strength and resistance to deformation than SMA and GA, thus providing better fatigue performance. However, it is worth noting that the influence of environmental factors such as processes of frost degradation of materials and penetration of chlorides from the ice melting agents as well as the transversal loading distribution coefficient on the fatigue life of the orthotropic steel–asphalt concrete composite deck were not considered in the study.

## 3. Conclusions

The number of papers published in this Special Issue of *Materials* demonstrates the interest attracted by the exploration of innovative materials for use in asphalt concrete pavements. The issues covered proved that the international scientific community is not only looking for innovative, but also sustainable materials, which meet the needs of the present without compromising the ability of future generations to meet their own needs. Recycled materials such as crumb rubber, renewable bio-materials and reclaimed asphalt, as well as graphite nanoplatelets and natural materials used as rejuvenators, tend toward the circular economy model, which aims at enhancing the resource efficiency by exploiting wastes that can be used as raw materials in different processes.

## Data Availability

Not applicable.
